# Recent Advances in the Analysis of PCBs and Pesticides in Human Adipose Tissue

**DOI:** 10.6028/jres.093.070

**Published:** 1988-06-01

**Authors:** James C. Peterson, Peter Robinson

**Affiliations:** Pacific Toxicology Laboratories, 1545 Pontius Avenue, Los Angeles, CA 90025

## Introduction

Due to their bioaccumulative and persistent nature, determination of PCB and organochlorine pesticide levels in human adipose tissue can be a useful biological indicator of exposure. The analytical methods previously used are relatively tedious, labor intensive and not readily amenable to automation. We have combined the use of sweep co-distillation cleanup [1] and high resolution gas chromatography with automated on-column injection to efficiently process and quantitate levels of these compounds in fat samples.

## Methods

Tissue samples (200 to 500 mg) are dried with sodium sulfate and extracted three times with petroleum ether. The solvent is totally removed and the 100 to 500 mg of the remaining extracted fat is ready for cleanup by sweep co-distillation (SGE, Australia). The principle of sweep co-distillation is based on the differential volatility of fat and the organochlorine compounds of interest and utilizes a process which is similar to a preparative gas chromatograph. The rendered or extracted fat is injected directly into a heated tube filled with uncoated glass beads. The pesticides and PCBs are carried through the column by nitrogen, leaving behind the fat. The analytes are retained on a silica gel trap at the end of the glass head column and are eluted from the trap in two solvent fractions.

The first fraction is eluted with 7 mL of hexane and contains PCBs, hexachlorobenzene, mirex and a portion of the heptachlor (50%) and p,p′-DDE (30%). Fraction II is eluted with 8 mL of 20% ethyl ether in hexane and contains p,p′-DDT, p,p′-DDD, p,p′-DDE (70%), trans-nonachlor, oxychlordane, *γ*-chlordane, *α*-chlordane, heptachlor (50%), heptachlor epoxide, lindane, *α*-BHC, *β*-BHC and Dieldrin. The two fractions are reduced to 2 mL each before GC analysis. This cleanup technique allows processing of 10 samples simultaneously, requires relatively little solvent and provides very clean extracts with less than 1% residual fat. The virtually fat free extracts reduce the problem of capillary column fouling when on-column injections are made.

The use of high resolution capillary columns for the separation of PCBs and organochlorine pesticides reduces possible interferences and allows quantitation of individual PCB congeners. This is vital for the analysis of human tissue where metabolism significantly changes the pattern of PCB congeners.

Automated on-column injection (Varian Model 3500, Walnut Creek, CA) allows unattended GC operation while providing the benefits of on-column injection including superior injection reproducibility which eliminates the need for an internal standard.

PCB congeners give different electron capture responses, generally increasing with the number of chlorines. Even isomers with the same number of chlorines often have widely varying response factors. Therefore, quantitation based on the assumption of equal response for all congeners in a particular Aroclor mixture will produce inaccurate results, especially in adipose tissue, where metabolism significantly changes the pattern of PCB congeners in a mixture.

In order to determine individual congener response factors, mean weight percents for all the major PCB peaks in each Aroclor mixture were determined by first analyzing the Aroclors by GC/MS (EI). Assuming that each congener responds equally in the GC/MS, the area percents of the PCB peaks will be approximately equal to the mean weight percent for those peaks. These mean weight percents are used to calculate the amounts of individual congeners in Aroclor standards of known concentration, which, in turn, are used to calculate corresponding ECD response factors. A few of these response factors have been satisfactorily validated analyzing available pure PCB congener standards (Ultra Chemicals, Hope, RI).

A control lipid sample was prepared by spiking rendered pork lard with 1 mg/kg of Aroclor 1260 and 0.10 to 0.30 mg/kg of each of the organochlorine pesticides.

## Results and Discussion

Quality assurance data collected over a 3-month period and representing 14 separate runs using the spiked lipid control (approximately once a week), indicates that this procedure produces excellent recoveries and precision (table 1). Each analyte, except for *β*-BHC (64%), has greater than a 78% recovery. PCBs, hexachlorobenzene and p,p′-DDE are recovered at slightly higher than 100%, most likely due to endogenous contamination in the lard used for spiking. Precision ranged from 8.3 to 13.5% relative standard deviation confirming the robust qualities of the method. Even p,p′-DDT which is the poorest performed in the sweep co-distillation process has satisfactory recoveries (78%) and precision (11.8%) indicating that p,p′-DDT breakdown is under control. Detection limits are 0.010 to 0.020 mg/kg for organochlorine pesticides and 0.050 mg/kg for PCBs.

Adipose tissue results from 136 analyzed human samples are presented in table 2. As evidenced by these results there are several ubiquitous analytes found in human adipose tissue. PCB levels were found in all samples analyzed and most closely resembled Aroclor 1260, with over 80% of the total congeners containing six or more chlorines. The relatively narrow concentration range of PCB, hexachlorobenzene, heptachlor epoxide, trans-nonachlor and oxychlordane residues indicates a similar exposure history to these compounds in the population.

On the other hand, Dieldrin, p,p′-DDT and its metabolite p,p′-DDE display a greater concentration variation suggesting individual differences in either exposure or metabolism. *α*-Chlordane, *γ*-chlordane and heptachlor were present in less than 1% of the individuals tested. However, their metabolites, oxychlordane and heptachlor epoxide, were always present. Similarly, lindane and *α*-BHC were rarely detected, hut a biorefractory contaminant of technical lindane, *β*-BHC, was always present.

## Figures and Tables

**Table 1:** Recoveries and precision of PCBs and organochlorine pesticides in a spiked pork lard control (14 separate runs over a 3-month period).

Analyte	% Recovery	CV(%)
PCB (Aroclor 1260)	112	8.3
Hexachlorobenzene	105	9.1
*α*-BHC	93	13.5
Lindane	95	10.8
*β*-BHC	64	12.5
trans-Nonachlor	86	12.5
Oxychlordane	87	11.1
Heptachlor epoxide	95	10.8
*γ*-Chlordane	93	11.5
*α*-Chlordane	94	11.5
p,p′-DDE	114	12.5
p,p′-DDT	78	11.8
Dieldrin	36	12.6

**Table 2:** Chlorinated hydrocarbon residues in 136 adipose tissue extracts analyzed

Analyte	Mean, mg/kg	Standard deviation	% of samples containing residues^a^
PCB	1.03	0.57	100
Hexachlorobenzene	0.048	0.027	100
*β*-BHC	0.087	0.061	100
Heptachlor epoxide	0.056	0.035	100
trans-Nonachlor	0.122	0.070	96
Oxychlordane	0.090	0.039	100
p,p′-DDE	1.82	1.35	100
p,p′-DDT	0.093	0.220	75
Dieldrin	0.055	0.066	98

a Present in less than 1%: *α*-chlordane, *γ*-chlordane, lindane, *α*-BHC.
